# Supplementary material for: Silicon mitigates the adverse effects of drought on Lolium perenne physiological, morphometric and anatomical characters
Source: PeerJ. 2025 Feb 12;13:e18944. doi: 10.7717/peerj.18944 (PMC11829632; doi:10.7717/peerj.18944)
Supplement: Supplemental Information 2 — Abbreviations: Fs – steady-state chlorophyll fluorescence yields (relative units), Fm′ – maximal fluorescence signal (relative units), ΦPSII – quantum efficiency of photosystem II (relative units), ETR – photosynthetic electron transport rate (μmol m–2 s–1), NBI – nitrogen balance index (Dualex units), Chl – content of chlorophyll (Dualex units), Flv – content of flavonols (Dualex units), RWC – relative water content (%), SN – number of shoots (pcs.), SDM – shoot dry mass (g plant–1), RDM – root dry mass (g plant–1), RD – average diameter (mm), RL – root length (m), RA – root area (cm2), SRL – specific root length (m g–1), R:S – ratio of the root mass to the shoot mass. [file peerj-13-18944-s002.docx]

**Table S1.** Results of ANOVA (p-values) presenting the interactions for analyzed variables: cultivars (A), water conditions (B), Si application (C) and DAT (D).

| .Interactions | F*_s_* | F*_m_*' | Φ_PSII_ | ETR | NBI | Chl | Flv | RWC | SN | SDM | RDM | RD | RL | RA | SRL | R:S ratio |
| --- | --- | --- | --- | --- | --- | --- | --- | --- | --- | --- | --- | --- | --- | --- | --- | --- |
| A x B | 0.185 | 0.000 | 0.001 | 0.000 | 0.016 | 0.084 | 0.029 | 0.000 | 0.004 | 0.002 | 0.206 | 0.006 | 0.000 | 0.000 | 0.128 | 0.019 |
| A x C | 0.424 | 0.017 | 0.080 | 0.247 | 0.001 | 0.560 | 0.479 | 0.650 | 0.653 | 0.119 | 0.002 | 0.002 | 0.000 | 0.000 | 0.320 | 0.571 |
| B x C | 0.711 | 0.278 | 0.132 | 0.016 | 0.749 | 0.000 | 0.002 | 0.001 | 0.649 | 0.071 | 0.065 | 0.000 | 0.659 | 0.397 | 0.034 | 0.000 |
| A x D | 0.000 | 0.000 | 0.003 | 0.000 | 0.000 | 0.127 | 0.000 | 0.000 | 0.000 | 0.010 | 0.000 | 0.000 | 0.000 | 0.000 | 0.000 | 0.000 |
| B x D | 0.000 | 0.000 | 0.000 | 0.000 | 0.000 | 0.000 | 0.000 | 0.000 | 0.000 | 0.000 | 0.000 | 0.000 | 0.000 | 0.000 | 0.000 | 0.000 |
| C x D | 0.171 | 0.522 | 0.012 | 0.012 | 0.000 | 0.000 | 0.000 | 0.000 | 0.594 | 0.026 | 0.144 | 0.000 | 0.242 | 0.587 | 0.000 | 0.000 |
| A x B x C | 0.556 | 0.697 | 0.317 | 0.292 | 0.000 | 0.125 | 0.006 | 0.017 | 0.926 | 0.186 | 0.001 | 0.015 | 0.000 | 0.000 | 0.001 | 0.002 |
| A x B x D | 0.000 | 0.001 | 0.000 | 0.000 | 0.000 | 0.000 | 0.061 | 0.000 | 0.001 | 0.005 | 0.000 | 0.000 | 0.000 | 0.000 | 0.000 | 0.000 |
| A x C x D | 0.515 | 0.111 | 0.408 | 0.052 | 0.000 | 0.037 | 0.019 | 0.052 | 0.312 | 0.045 | 0.167 | 0.000 | 0.000 | 0.000 | 0.772 | 0.006 |
| B x C x D | 0.858 | 0.036 | 0.019 | 0.268 | 0.000 | 0.000 | 0.362 | 0.000 | 0.348 | 0.019 | 0.115 | 0.000 | 0.000 | 0.720 | 0.000 | 0.000 |
| A x B x C x D | 0.121 | 0.014 | 0.094 | 0.133 | 0.000 | 0.005 | 0.000 | 0.006 | 0.308 | 0.034 | 0.373 | 0.003 | 0.000 | 0.000 | 0.034 | 0.000 |

Abbreviations: F*_s_*– steady-state chlorophyll fluorescence yields (relative units), F*_m_*'– maximal fluorescence signal (relative units), Φ_PSII_– quantum efficiency of photosystem II (relative units), ETR– photosynthetic electron transport rate (μmol m^–2^ s^–1^), NBI – nitrogen balance index (Dualex units), Chl – content of chlorophyll (Dualex units), Flv – content of flavonols (Dualex units), RWC – relative water content (%), SN – number of shoots (pcs.), SDM – shoot dry mass (g plant^–1^), RDM – root dry mass (g plant^–1^), RD – average diameter (mm), RL – root length (m), RA – root area (cm^2^), SRL – specific root length (m g^–1^), R:S – ratio of the root mass to the shoot mass.
