# Supplementary material for: Silicon mitigates the adverse effects of drought on Lolium perenne physiological, morphometric and anatomical characters
Source: PeerJ. 2025 Feb 12;13:e18944. doi: 10.7717/peerj.18944 (PMC11829632; doi:10.7717/peerj.18944)
Supplement: Supplemental Information 3 — The mean values of features in columns, for individual terms, marked with the same lower-case letters did not differ significantly at p ≤ 0.05 Abbreviations: Fs – steady-state chlorophyll fluorescence yields (relative units), Fm′ – maximal fluorescence signal (relative units), ΦPSII – quantum efficiency of photosystem II (relative units), ETR – photosynthetic electron transport rate (μmol m–2 s–1), NBI – nitrogen balance index (Dualex units), Chl – content of chlorophyll (Dualex units), Flv – content of flavonols (Dualex units), RWC – relative water content (%), SN – number of shoots (pcs.), SDM – shoot dry mass (g plant–1), RDM – root dry mass (g plant–1), RD – average diameter (mm), RL – root length (m), RA – root area (cm2), SRL – specific root length (m g–1), R:S – ratio of the root mass to the shoot mass. [file peerj-13-18944-s003.docx]

**Table S2.** Detailed comparisons of means for individual terms (7, 14, 21 and 35 DAT).

| Cvs | Water conditions | Si application | F*_s_* | F*_m_'* | Φ_PSII_ | ETR | NBI | Chl | Flv | RWC | SN | SDM | RDM | RD | RL | RA | SRL | R:S ratio |
| --- | --- | --- | --- | --- | --- | --- | --- | --- | --- | --- | --- | --- | --- | --- | --- | --- | --- | --- |
|  |  |  | 7 DAT | | | | | | | | | | | | | | | |
| Bokser | control | Si- | 674^ab^ | 2263^a^ | 0.694^a^ | 84.9^b^ | 64.8^bc^ | 40.6^b^ | 0.64^a^ | 87.0^a^ | 14.4^c^ | 0.069^ab^ | 0.073^d^ | 0.486^c^ | 4.29^cd^ | 64.5^c^ | 58.7^a^ | 1.06^b^ |
|  |  | Si+ | 642^ab^ | 2119^a^ | 0.695^a^ | 85.0^b^ | 73.2^c^ | 42.9^b^ | 0.59^a^ | 93.1^b^ | 13.8^bc^ | 0.073^b^ | 0.069^d^ | 0.458^abc^ | 4.95^d^ | 71.3^c^ | 71.6^a^ | 0.95^ab^ |
|  | drought | Si- | 675^ab^ | 1980^a^ | 0.655^a^ | 67.9^a^ | 59.7^ab^ | 42.2^b^ | 0.76^b^ | 87.0^a^ | 12.6^abc^ | 0.062^ab^ | 0.051^abc^ | 0.632^d^ | 2.90^a^ | 59.7^bc^ | 56.9^a^ | 0.77^a^ |
|  |  | Si+ | 710^b^ | 2076^a^ | 0.656^a^ | 64.6^a^ | 59.3^ab^ | 39.3^ab^ | 0.63^a^ | 89.1^ab^ | 12.1^abc^ | 0.069^ab^ | 0.071^d^ | 0.476^bc^ | 4.20^bcd^ | 62.^5c^ | 59.6^a^ | 1.03^b^ |
| Stadion | control | Si- | 641^ab^ | 2184^a^ | 0.705^a^ | 84.8^b^ | 60.0^ab^ | 42.7^b^ | 0.67^ab^ | 87.4^a^ | 12.0^abc^ | 0.055^ab^ | 0.041^a^ | 0.427^abc^ | 2.90^a^ | 38.9^a^ | 71.7^a^ | 0.74^a^ |
|  |  | Si+ | 664^ab^ | 2179^a^ | 0.693^a^ | 80.5^b^ | 65.7^bc^ | 40.7^b^ | 0.62^a^ | 88.4^a^ | 12.7^abc^ | 0.059^ab^ | 0.047^ab^ | 0.418^ab^ | 3.35^abc^ | 42.9^ab^ | 71.7^a^ | 0.79^a^ |
|  | drought | Si- | 621^ab^ | 2062^a^ | 0.698^a^ | 79.3^b^ | 62.4^ab^ | 40.7^b^ | 0.69^ab^ | 86.4^a^ | 10.8^ab^ | 0.054^a^ | 0.061^bcd^ | 0.411^a^ | 3.27^ab^ | 42.1^ab^ | 54.5^a^ | 1.07^b^ |
|  |  | Si+ | 567^a^ | 2187^a^ | 0.692^a^ | 85.2^b^ | 53.9^a^ | 36.5^a^ | 0.67^ab^ | 85.9^a^ | 10.1^a^ | 0.063^ab^ | 0.068^cd^ | 0.419^bc^ | 4.12^bcd^ | 54.2^abc^ | 66.2^a^ | 1.10^b^ |
|  |  |  | 14 DAT | | | | | | | | | | | | | | | |
| Bokser | control | Si- | 751^b^ | 2249^b^ | 0.672^c^ | 84.0^c^ | 65.1^c^ | 40.9^c^ | 0.63^b^ | 90.3^c^ | 23.5^bc^ | 0.156^b^ | 0.134^b^ | 0.344^a^ | 9.87^b^ | 108.7^b^ | 66.8^b^ | 0.86^ab^ |
|  |  | Si+ | 755^b^ | 2408^b^ | 0.691^c^ | 88.1^c^ | 91.8^d^ | 35.8^bc^ | 0.40^a^ | 94.6^c^ | 24.7^c^ | 0.167^b^ | 0.132^b^ | 0.378^a^ | 11.45^b^ | 132.7^bc^ | 87.2^c^ | 0.79^ab^ |
|  | drought | Si- | 556^b^ | 714^a^ | 0.148^a^ | 15.4^a^ | 32.7^a^ | 26.4^a^ | 0.84^c^ | 51.8^b^ | 11.7^a^ | 0.070^a^ | 0.062^a^ | 0.372^a^ | 4.23^a^ | 48.7^a^ | 69.1^b^ | 0.88^ab^ |
|  |  | Si+ | 461^a^ | 571^a^ | 0.185^a^ | 22.4^b^ | 40.0^ab^ | 37.8^bc^ | 0.78^c^ | 49.5^ab^ | 10.4^a^ | 0.076^a^ | 0.088^a^ | 0.360^a^ | 4.56^a^ | 63.3^a^ | 52.2^a^ | 1.30^c^ |
| Stadion | control | Si- | 745^b^ | 2187^b^ | 0.656^c^ | 82.2^c^ | 57.2^c^ | 34.6^b^ | 0.63^b^ | 87.7^c^ | 19.6^b^ | 0.178^b^ | 0.114^b^ | 0.328^a^ | 10.50^b^ | 108.1^b^ | 83.6^c^ | 0.64^a^ |
|  |  | Si+ | 747^b^ | 2233^b^ | 0.662^c^ | 83.2^c^ | 58.3^c^ | 34.9^bc^ | 0.60^b^ | 86.3^c^ | 20.3^b^ | 0.176^b^ | 0.180^c^ | 0.362^a^ | 15.21^c^ | 169.1^c^ | 93.8^c^ | 1.02^bc^ |
|  | drought | Si- | 429^a^ | 494^a^ | 0.199^ab^ | 20.4^ab^ | 33.2^a^ | 25.8^a^ | 1.20^d^ | 42.4^a^ | 10.8^a^ | 0.068^a^ | 0.068^a^ | 0.361^a^ | 5.01^a^ | 56.9^a^ | 69.6^b^ | 1.02^bc^ |
|  |  | Si+ | 458^a^ | 664^a^ | 0.285^b^ | 26.0^b^ | 41.9^b^ | 38.0^bc^ | 0.81^c^ | 53.6^b^ | 11.5^a^ | 0.078^a^ | 0.078^a^ | 0.366^a^ | 5.13^a^ | 61.0^a^ | 69.1^b^ | 1.00^b^ |
|  |  |  | 21 DAT | | | | | | | | | | | | | | | |
| Bokser | control | Si- | 749^c^ | 2467^cd^ | 0.683^bc^ | 85.5^b^ | 49.1^bc^ | 35.8^c^ | 0.71^abc^ | 86.5^c^ | 39.5^c^ | 0.651^b^ | 0.424^d^ | 0.381^ab^ | 36.37^d^ | 389.1^d^ | 86.2^ab^ | 0.65^b^ |
|  |  | Si+ | 697^c^ | 2246^b^ | 0.681^bc^ | 85.2^b^ | 41.9^b^ | 29.1^b^ | 0.73^abcd^ | 87.0^c^ | 38.7^c^ | 0.664^b^ | 0.410^d^ | 0.349^a^ | 35.28^d^ | 350.4^d^ | 86.2^ab^ | 0.67^b^ |
|  | drought | Si- | 429^ab^ | 457^a^ | 0.008^a^ | 0.4^a^ | 12.7^a^ | 10.4^a^ | 0.81^cd^ | 28.6^ab^ | 11.0^a^ | 0.029^a^ | 0.064^a^ | 0.342^a^ | 6.46^a^ | 75.6^ab^ | 100.6^b^ | 2.18^c^ |
|  |  | Si+ | 332^a^ | 337^a^ | 0.004^a^ | 0.4^a^ | 15.9^a^ | 13.4^a^ | 0.78^cd^ | 27.2^a^ | 11.7^a^ | 0.029^a^ | 0.079^a^ | 0.355^a^ | 5.94^a^ | 67.5^a^ | 75.6^a^ | 2.69^d^ |
| Stadion | control | Si- | 820^c^ | 2325^bc^ | 0.646^b^ | 79.9^b^ | 55.1^c^ | 35.4^c^ | 0.65^ab^ | 86.6^c^ | 29.4^b^ | 0.564^b^ | 0.198^b^ | 0.430^c^ | 13.32^b^ | 173.2^bc^ | 78.2^a^ | 0.36^a^ |
|  |  | Si+ | 762^c^ | 2558^d^ | 0.701^c^ | 81.7^b^ | 44.4^b^ | 29.2^b^ | 0.62^a^ | 85.7^c^ | 23.7^b^ | 0.570^b^ | 0.297^c^ | 0.403^bc^ | 19.52^c^ | 226.5^c^ | 69.6^a^ | 0.52^ab^ |
|  | drought | Si- | 506^b^ | 509^a^ | 0.004^a^ | 0.4^a^ | 11.2^a^ | 11.0^a^ | 0.85^d^ | 31.7^ab^ | 9.2^a^ | 0.032^a^ | 0.058^a^ | 0.366^ab^ | 4.50^a^ | 52.4^a^ | 77.9^a^ | 1.93^c^ |
|  |  | Si+ | 483^ab^ | 485^a^ | 0.004^a^ | 0.5^a^ | 15.9^a^ | 14.8^a^ | 0.80^cd^ | 34.5^b^ | 9.6^a^ | 0.037^a^ | 0.089^a^ | 0.364^ab^ | 6.24^a^ | 70.7^a^ | 70.9^a^ | 2.65^d^ |
|  |  |  | 35 DAT | | | | | | | | | | | | | | | |
| Bokser | control | Si- | 999^c^ | 2349^bc^ | 0.633^a^ | 79.0^cd^ | 22.9^b^ | 19.5^c^ | 0.98^c^ | 83.9^d^ | 52.0^e^ | 0.755^c^ | 1.110^b^ | 0.387^ab^ | 138.49^d^ | 1639.5^d^ | 114.4^c^ | 1.47^ab^ |
|  |  | Si+ | 1012^c^ | 2320^bc^ | 0.624^a^ | 83.6^d^ | 22.5^b^ | 19.6^c^ | 0.94^c^ | 85.3^d^ | 47.5d^e^ | 0.770^c^ | 1.124^b^ | 0.404^ab^ | 120.80^c^ | 1415.8^c^ | 107.5^bc^ | 1.47^ab^ |
|  | drought | Si- | 466^a^ | 1499^a^ | 0.700^b^ | 42.0^a^ | 26.3^bc^ | 14.0^b^ | 0.59^ab^ | 38.9^a^ | 18.6^c^ | 0.074^a^ | 0.098^a^ | 0.428^b^ | 7.97^a^ | 115.3^a^ | 81.3^a^ | 1.23^a^ |
|  |  | Si+ | 476^ab^ | 1555^a^ | 0.694^b^ | 54.5^b^ | 25.5^bc^ | 14.9^b^ | 0.47^a^ | 57.3^b^ | 17.2^bc^ | 0.079^a^ | 0.114^a^ | 0.367^a^ | 10.51^a^ | 126.8^a^ | 92.8^ab^ | 1.56^ab^ |
| Stadion | control | Si- | 978^c^ | 2618^c^ | 0.627^a^ | 81.4^d^ | 29.5^c^ | 24.3^d^ | 0.78^bc^ | 84.1^d^ | 37.4^d^ | 0.599^b^ | 1.186^bc^ | 0.387^ab^ | 104.18^b^ | 1257.6^b^ | 88.0^a^ | 2.11^d^ |
|  |  | Si+ | 963^c^ | 2470^bc^ | 0.613^a^ | 79.4^cd^ | 25.1^bc^ | 20.0^c^ | 0.88^c^ | 84.2^d^ | 39.3^d^ | 0.774^c^ | 1.332^c^ | 0.403^ab^ | 119.27^c^ | 1523.2^cd^ | 89.6^a^ | 1.77^bc^ |
|  | drought | Si- | 644^ab^ | 2163^b^ | 0.651^ab^ | 68.6^c^ | 16.3^a^ | 6.5^a^ | 0.45^a^ | 57.2^b^ | 5.3^ab^ | 0.067^a^ | 0.118^a^ | 0.435^b^ | 9.74^a^ | 122.2^a^ | 83.3^a^ | 1.76^bc^ |
|  |  | Si+ | 662^b^ | 2277^bc^ | 0.697^b^ | 72.5^cd^ | 24.2^bc^ | 12.9^b^ | 0.40^a^ | 69.9^c^ | 4.0^a^ | 0.072^a^ | 0.125^a^ | 0.392^ab^ | 11.51^a^ | 138.5^a^ | 94.3^ab^ | 1.93^cd^ |

The mean values of features in columns for individual terms marked with the same lower-case letters did not differ significantly at p≤0.05.

Abbreviations: F*_s_*– steady-state chlorophyll fluorescence yields (relative units), F*_m_*'– maximal fluorescence signal (relative units), Φ_PSII_– quantum efficiency of photosystem II (relative units), ETR– photosynthetic electron transport rate (μmol m^–2^ s^–1^), NBI – nitrogen balance index (Dualex units), Chl – content of chlorophyll (Dualex units), Flv – content of flavonols (Dualex units), RWC – relative water content (%), SN – number of shoots (pcs.), SDM – shoot dry mass (g plant^–1^), RDM – root dry mass (g plant^–1^), RD – average diameter (mm), RL – root length (m), RA – root area (cm^2^), SRL – specific root length (m g^–1^), R:S – ratio of the root mass to the shoot mass.
